# Supplementary material for: NetQuilt: deep multispecies network-based protein function prediction using homology-informed network similarity
Source: Bioinformatics. 2021 Feb 12;37(16):2414–22. doi: 10.1093/bioinformatics/btab098 (PMC8388039; doi:10.1093/bioinformatics/btab098)
Supplement: btab098_Supplementary_Datay [file btab098_supplementary_datay.pdf]

Supplementary Material
NetQuilt: Deep Multispecies Network-based Protein Function Prediction using Homology-informed Network Similarity

Meet Barot[1], Vladimir Gligorijević[2], Kyunghyun Cho[1], and Richard Bonneau[1,2]

[1]Center for Data Science, New York University, New York, NY, USA
[2]Center for Computational Biology, Flatiron Institute, New York, NY, USA

1 Network and Annotations – Eukaryotes

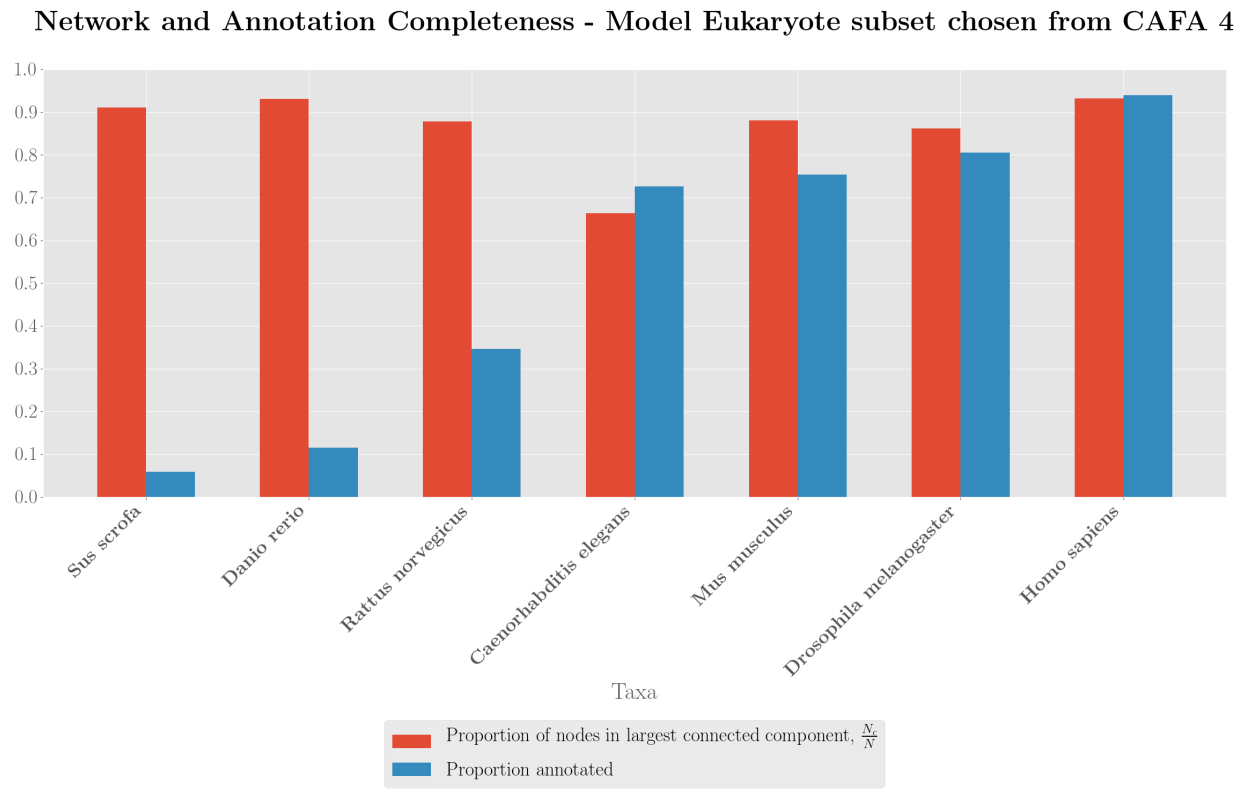

Figure 1: Proportions of STRING proteins of a given Eukaryote taxonomy ID that are annotated in at least one branch of the Gene Ontology with any evidence code and proportions that are in the STRING experimental network's largest connected component, sorted by the sum of the two proportions of each species.

2 Network and Annotations – Bacteria

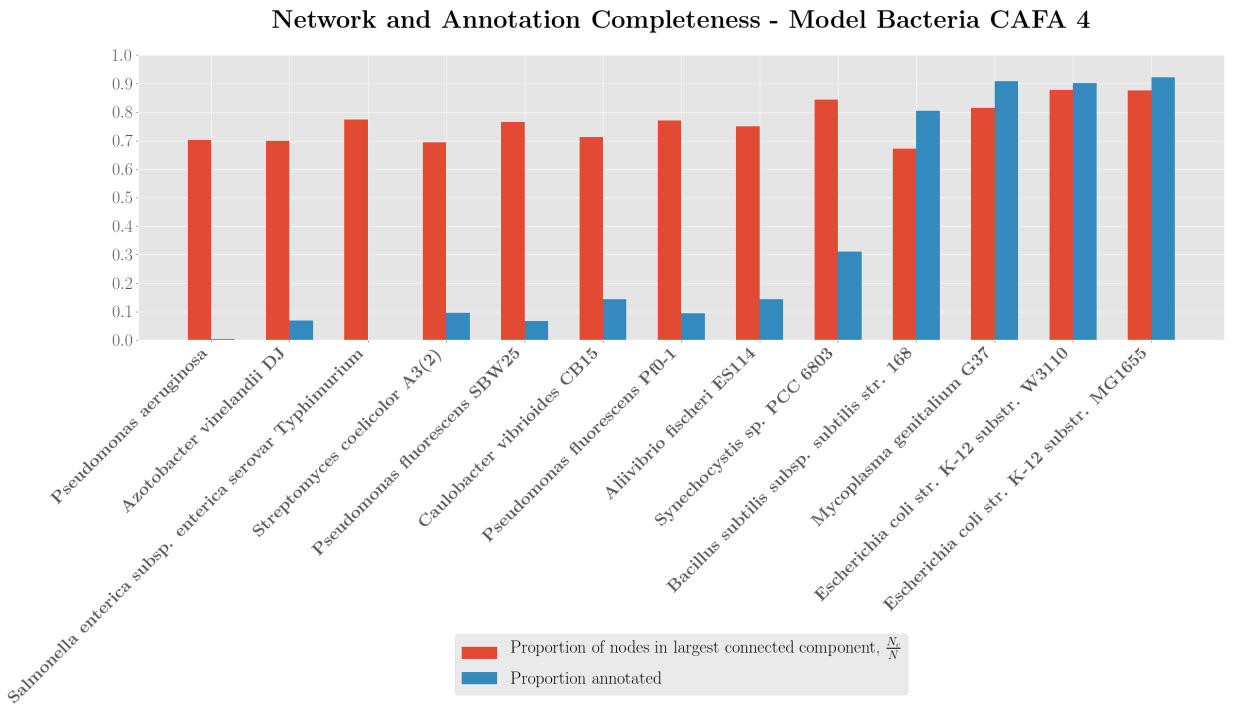

Figure 2: Proportions of STRING proteins of a given Bacteria taxonomy ID that are annotated in at least one branch of the Gene Ontology with any evidence code and proportions that are in the STRING experimental network's largest connected component, sorted by the sum of the two proportions of each species.

3 Experimental PPI Network statistics

Table 1: **Network statistics for Chosen CAFA 4 Eukaryote Experimental PPI networks**. $\frac{N_c}{N}$ refers to the ratio of largest connected component nodes to the total number of nodes in the graph.

**Scientific name**	**Taxonomy ID**	**Nodes**	**Edges**	$\mathbf{\frac{N_c}{N}}$
C. elegans	6239	18181	1044754	0.66289
D. melanogaster	7227	13046	749503	0.861873
D. rerio	7955	24681	4198604	0.93104
H. sapiens	9606	19354	2287192	0.931745
S. scrofa	9823	21284	2892886	0.910449
M. musculus	10090	21291	2739040	0.880607
R. norvegicus	10116	22234	3374903	0.878205

Table 2: **Network Statistics for CAFA 4 Bacteria Experimental PPI Networks.** $\frac{N_c}{N}$ refers to the ratio of largest connected component nodes to the total number of nodes in the graph.

**Scientific name**	**Taxonomy ID**	**Nodes**	**Edges**	$\mathbf{\frac{N_c}{N}}$
P. aeruginosa	287	6267	161805	0.703048
E. coli str. K-12 substr. MG1655	511145	4125	66075	0.876848
E. coli str. K-12 substr. W3110	316407	4210	61533	0.87886
S. enterica subsp. enterica serovar Ty...	90371	4418	59412	0.774785
M. genitalium G37	243273	474	7361	0.814346
B. subtilis subsp. subtilis str. 168	224308	4181	69451	0.672088
C. vibrioides CB15	190650	3721	82083	0.713518
A. fischeri ES114	312309	3797	74238	0.749802
Synechocystis sp. PCC 6803	1148	3167	59624	0.843701
P. fluorescens SBW25	216595	5881	245532	0.766026
P. fluorescens Pf0-1	205922	5681	225074	0.770111
A. vinelandii DJ	322710	4955	115245	0.699697
S. coelicolor A3(2)	100226	7741	224300	0.694871

4 Gene Ontology Terms Considered

Table 3: **Number of GO terms considered for each organism and branch.** GO terms were selected for organisms based on the percentage of the proteome annotated by a possible GO term. A GO term must annotate between 0.5% - 5% of the organism's proteome to be part of the evaluation.

	Molecular Function	Biological Process	Cellular Component
E. coli	196	509	46
Human	242	1458	232
Fly	195	962	178
Mouse	252	1486	234

5 Hyperparameter search

The hyperparameters for our models were chosen as follows: We define the search space for hyperparameters that included the values of the architecture described in [1]. For each experiment, we sample 1% of the search space defined by the sets of values given in the columns of the table below. After an experiment, the sets of values were changed based on the hyperparameter settings' correlation with validation F-measure of the previous experiment.

Table 4: Hyperparameter value sets for each experiment. Each column indicates the search space of possible hyperparameter values to define an architecture. "None" values for Maxout Units refer to ReLU being used instead of maxout activation.

Experiment	1	2	3
Hidden Dim 1	{100, 500, 0}	{500, 1000, 0}	{500, 1000}
Hidden Dim 2	{200, 700, 0}	{200, 700, 0}	{200, 700, 0}
Hidden Dim 3	{300, 800, 0}	{300, 800, 0}	{300, 800}
Hidden Dim 4	{500, 1000, 0}	{500, 1000, 0}	{800, 1000, 1500}
Maxout Units	{None, 3, 5}	{None, 3}	{3, 4, 5}
Dropout	{0.25, 0.5, 0.75}	{0.25, 0.5}	{0.2, 0.3, 0.4, 0.5}
Epochs	{100, 150, 200}	{150, 200, 250}	{100, 250, 300}
Learning Rate	{0.01, 0.05}	{0.01}	{0.01}
Batch Size	{16, 32, 64}	{16, 32, 64}	{16, 32}

All models were trained and evaluated with cross-validation on a dataset consisting of PPI networks that had at least 20% of proteins annotated. Note: this was not the same dataset used for the final evaluations, on which we report performance.
Dataset organisms:
Eukaryotes: S. pombe, S. cerevisiae, H. sapiens, M. musculus, D. melanogaster, A. thaliana, C. elegans, R. norvegicus
Bacteria: E. coli K-12 substrains MG1655, W3110 and DH10B, B. subtilis strain 168, H. influenzae Rd KW20, M. genitalium G37

6 Leave-one-species-out validation (human and mouse)

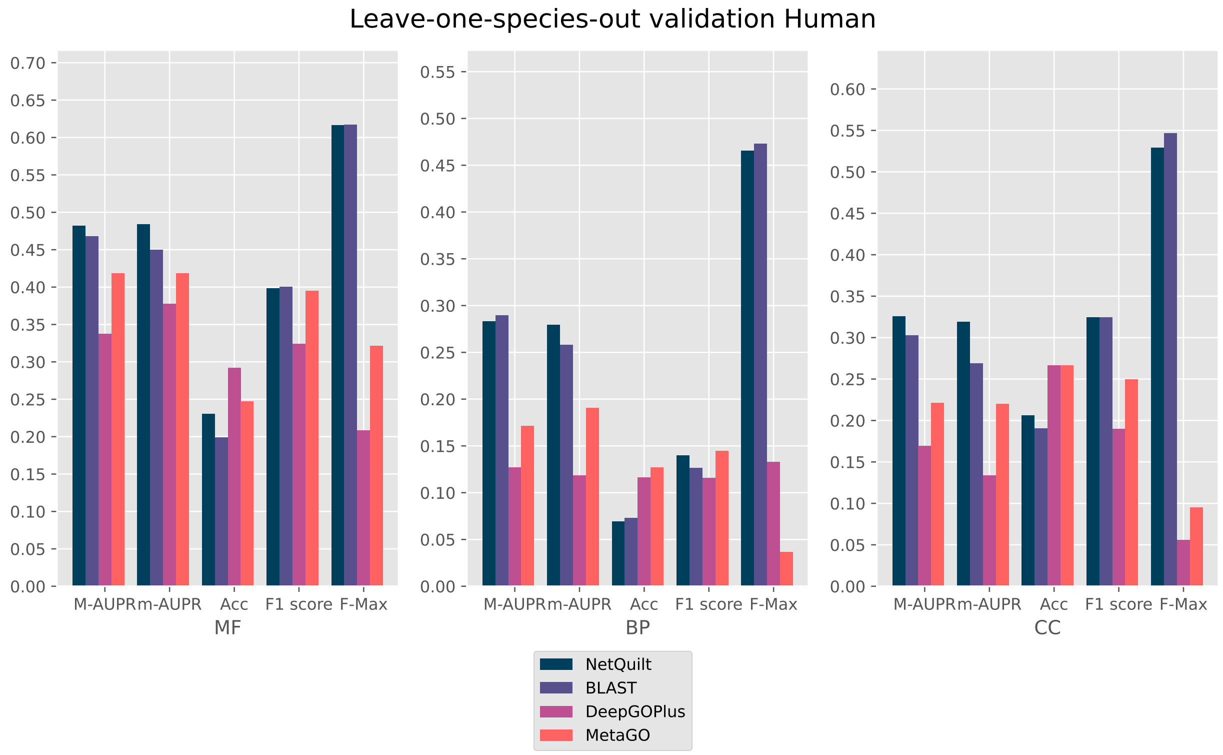

Figure 3: **H. sapiens** annotations.

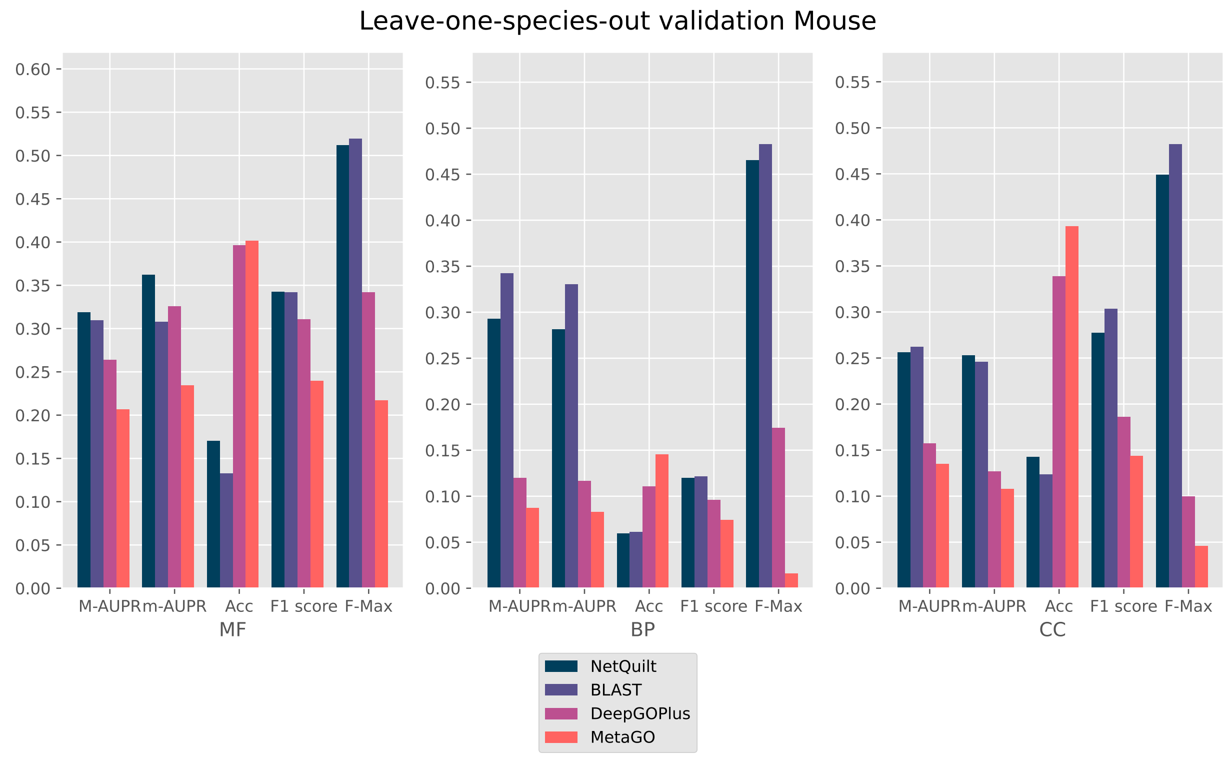

Figure 4: **M. musculus** annotations.

7 α search

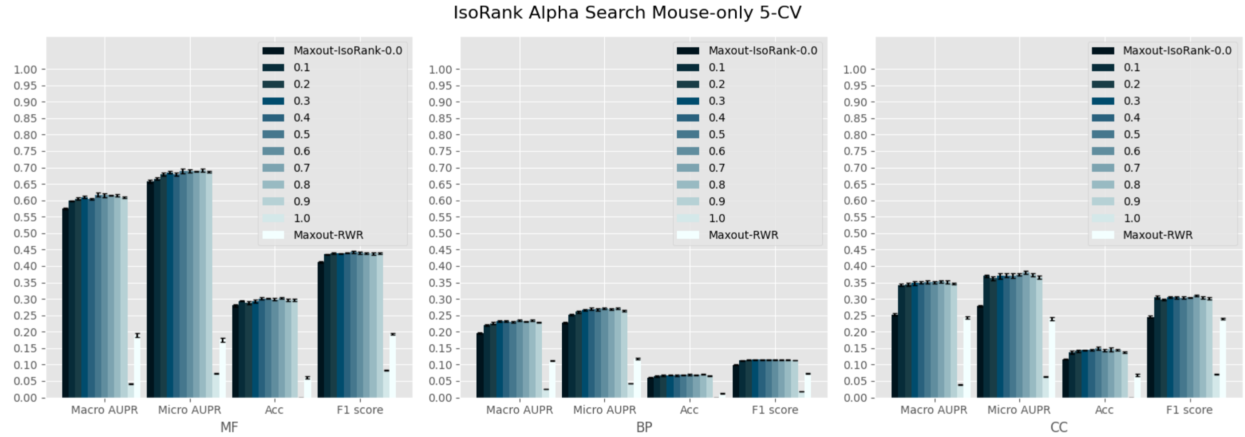

Figure 5: **M. musculus** annotations, cross validation with 5 trials. Maxout-IsoRank-0.0 (and all other bars labeled with numbers) refer to using the mouse IsoRank matrix only, with different values of α, as features. Maxout-RWR refers to a maxout neural network trained on a random-walk-with-restarts matrix computed for the mouse PPI network (this method, as well as the IsoRank $\alpha = 1$ setting, creates features with no contribution from homology).

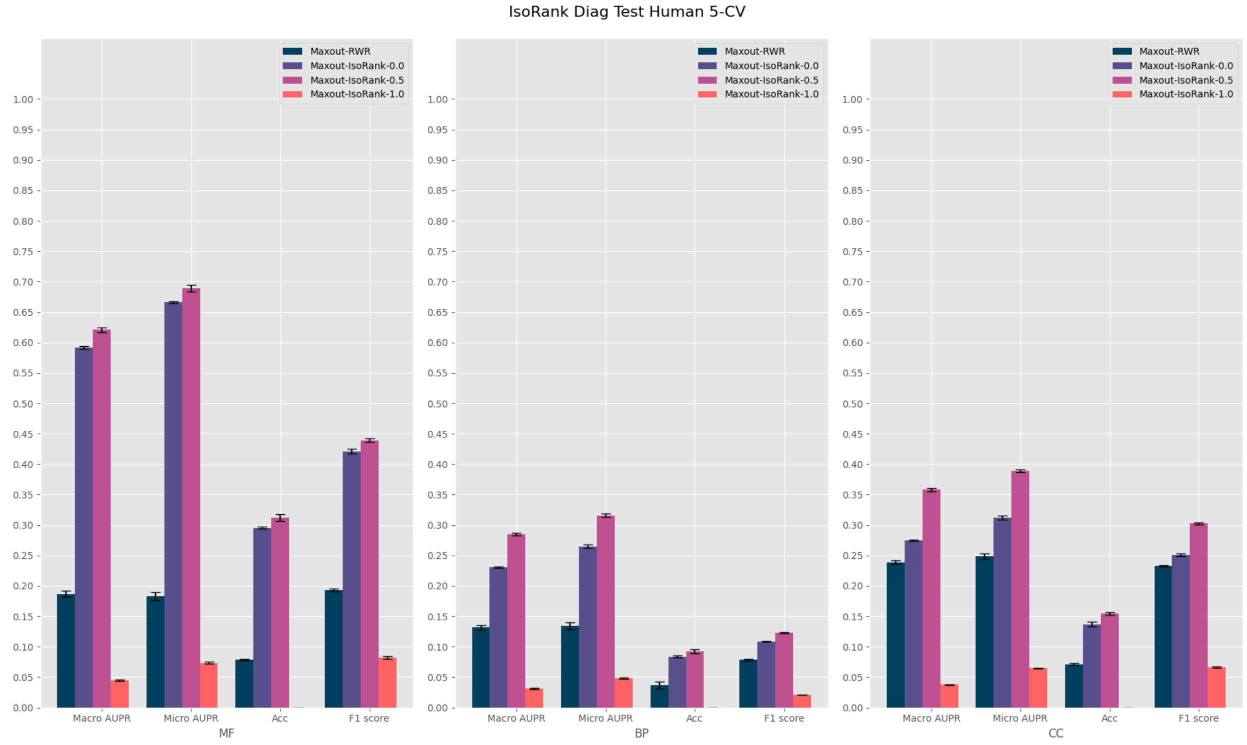

Figure 6: **H. sapiens** annotations and mouse IsoRank matrix only, cross validation with 5 trials. Maxout-RWR refers to a maxout neural network trained on a random-walk-with-restarts matrix computed for the mouse PPI network (this method, as well as the IsoRank $\alpha = 1$ setting, creates features with no contribution from homology). $\alpha = 0.5$ was chosen for all eukaryote experiments as a result of the α search in Supplemental Figure 5.

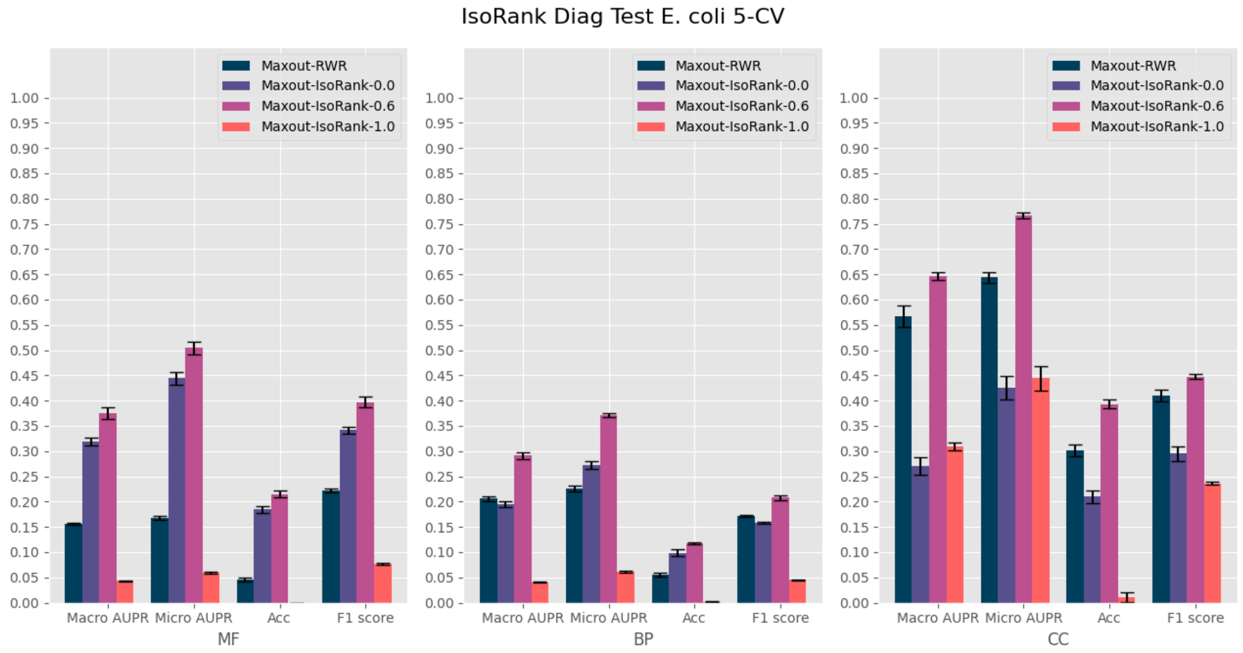

Figure 7: **E. coli** annotations and E. coli IsoRank matrix only, cross validation with 5 trials. Maxout-RWR refers to a maxout neural network trained on a random-walk-with-restarts matrix computed for the mouse PPI network (this method, as well as the IsoRank $\alpha = 1$ setting, creates features with no contribution from homology).

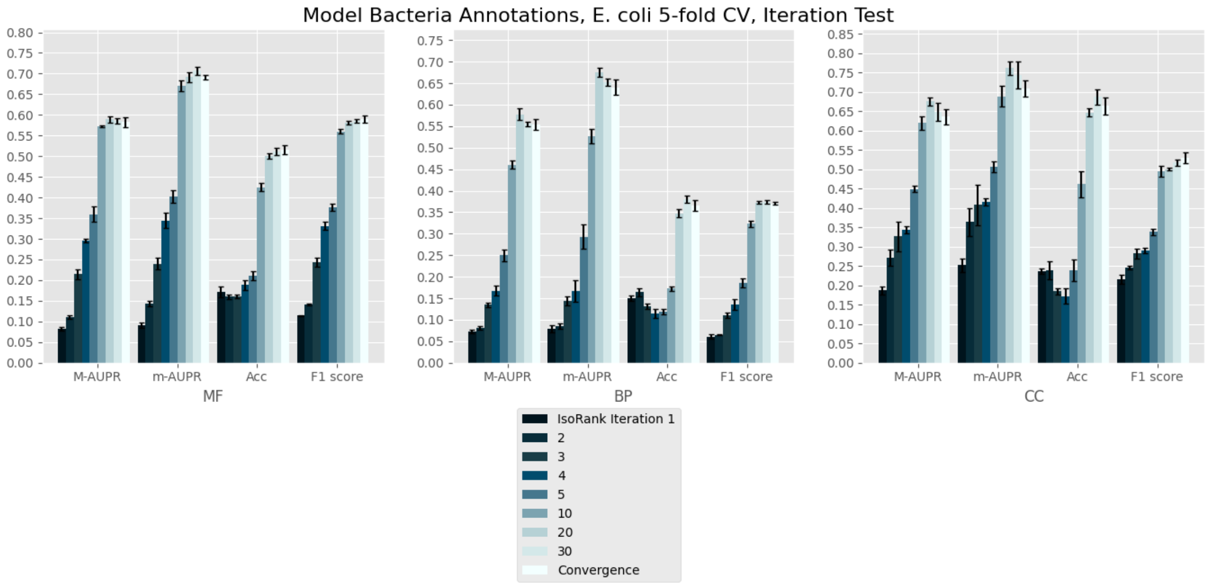

Figure 8: 5 trial CV on E. coli, trained with CAFA bacteria annotations. Test of how performance changes with different iterations of IsoRank.

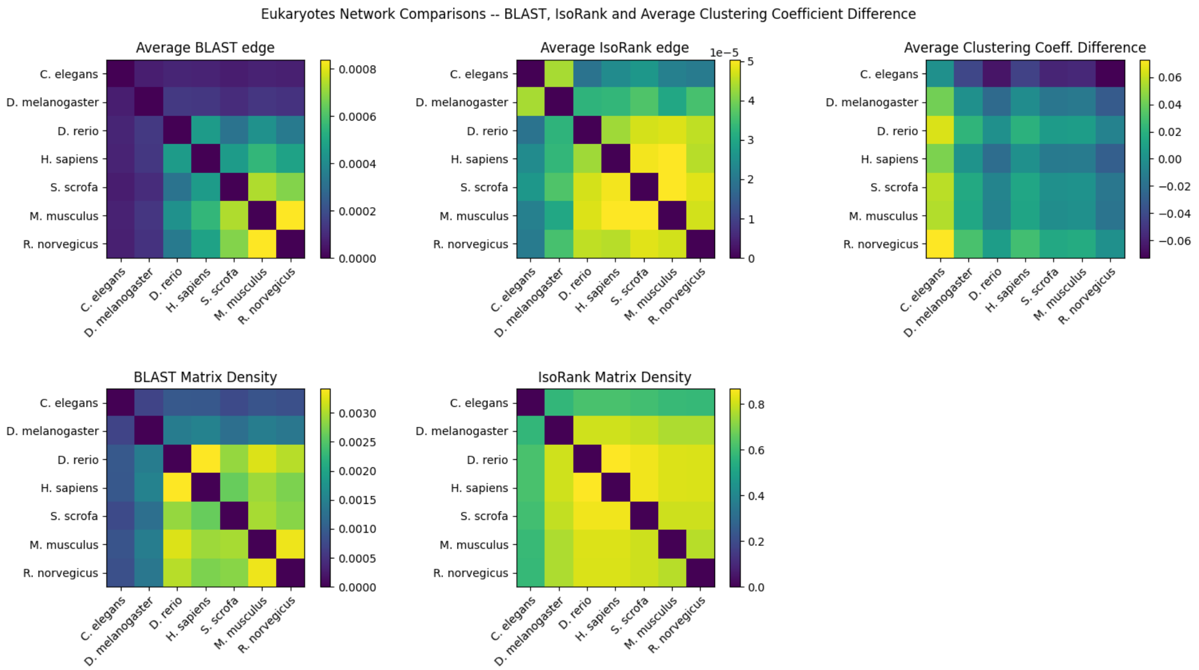

Figure 9: Eukaryote network comparisons. Average BLAST edge refers to the average negative log e-value between the proteins of the two compared organisms (with e-value threshold 10^{-3}), IsoRank edge is the average element of the IsoRank matrix between the two organisms, and the Average Clustering Coeff. Difference is the difference between the average clustering coefficients of the two compared organisms. Since the clustering coefficient difference can be negative, the plot is not symmetric. Additionally, the diagonal values are not computed for the other plots.

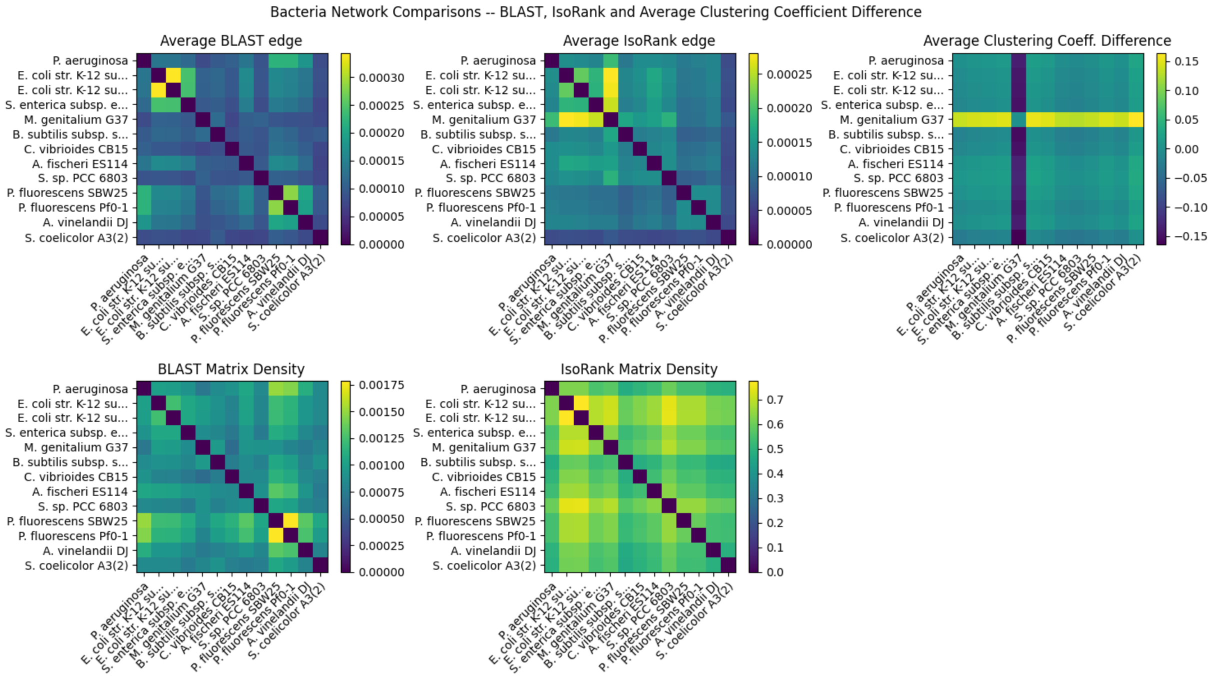

Figure 10: Bacterial network comparisons. Average BLAST edge refers to the average negative log e-value between the proteins of the two compared organisms (with e-value threshold 10^{-3}), IsoRank edge is the average element of the IsoRank matrix between the two organisms, and the Average Clustering Coeff. Difference is the difference between the average clustering coefficients of the two compared organisms. Since the clustering coefficient difference can be negative, the plot is not symmetric. Additionally, the diagonal values are not computed for the other plots.

References

[1] Cen Wan, Domenico Cozzetto, Rui Fa, and David T Jones. Using deep maxout neural networks to improve the accuracy of function prediction from protein interaction networks. *PloS one*, 14(7), 2019.
